# Randomized, Double-blinded, Placebo-controlled Trial of Amoxicillin/Clavulanic Acid to Prevent Preterm Delivery in Twin Gestation

**DOI:** 10.1155/S1064744995000512

**Published:** 1995

**Authors:** Mark T. Peters, Charles E. L. Brown, Audrey Baum, Rick Risser

**Affiliations:** 1 Department of Obstetrics and Gynecology University of Texas Southwestern Medical Center at Dallas Dallas TX USA; 2 Department of Academic Computing University of Texas Southwestern Medical Center at Dallas Dallas TX USA; 3 Department of Obstetrics and Gynecology 319 West, 1600 South Andrews Avenue Fort Lauderdale FL 33316 USA

## Abstract

*Objective:* The objective of this study was to determine whether prophylactic treatment with oral broad-spectrum antimicrobial therapy improves pregnancy outcomes in twin gestations.

*Methods:* Patients with twin gestations between 24 and 32 weeks were randomized to receive amoxicillin/clavulanic acid or placebo. Those patients randomized before 24 weeks received a 1-week course at 24 and at 28 weeks gestation. Those patients entered later than 24 weeks received a 1-week course either at 28 weeks or at enrollment (up to 32 weeks). Other than antibiotic use, the management of the groups was identical and unchanged from the routine care of twin gestations.

Results: Of 149 twin pregnancies enrolled, 76 were randomized to the drug group and 73 to the placebo group. There was no significant difference in mean gestational age at delivery (35.9 vs. 35.7 weeks), birth weight (2,358 vs. 2,344 g), mean neonatal nursery stay (9.9 vs. 11.7 days), or respiratory distress syndrome (6/76 vs. 4/73) in the drug vs. placebo group, respectively.

*Conclusions:* The addition of prophylactic oral broad-spectrum antimicrobial therapy to the standard antepartum management of twin gestations had no demonstrable effect on the gestational age at delivery, birth weight, or neonatal complications. There did not appear to be any beneficial effect of the prophylactic use of amoxicillin/clavulanic acid in this clinical setting.


Infectious Diseases in Obstetrics and Gynecology 3:158–163 (1995)
© 1995 Wiley-Liss, Inc.

### Randomized, Double-Blinded, Placebo-Controlled Trial of Amoxicillin/Clavulanic Acid to Prevent Preterm Delivery in Twin Gestation

**Mark T. Peters, Charles E.L. Brown, Audrey Baum, and Rick Risser**

*Departments of Obstetrics and Gynecology (M.T.P., C.E.L.B., A.B.) and Academic Computing (R.R.), University of Texas Southwestern Medical Center at Dallas, Dallas, TX*

### Abstract

***Objective:*** The objective of this study was to determine whether prophylactic treatment with oral broad-spectrum antimicrobial therapy improves pregnancy outcomes in twin gestations.

***Methods:*** Patients with twin gestations between 24 and 32 weeks were randomized to receive amoxicillin/clavulanic acid or placebo. Those patients randomized before 24 weeks received a 1-week course at 24 and at 28 weeks gestation. Those patients entered later than 24 weeks received a 1-week course either at 28 weeks or at enrollment (up to 32 weeks). Other than antibiotic use, the management of the groups was identical and unchanged from the routine care of twin gestations.

***Results:*** Of 149 twin pregnancies enrolled, 76 were randomized to the drug group and 73 to the placebo group. There was no significant difference in mean gestational age at delivery (35.9 vs. 35.7 weeks), birth weight (2,358 vs. 2,344 g), mean neonatal nursery stay (9.9 vs. 11.7 days), or respiratory distress syndrome (6/76 vs. 4/73) in the drug vs. placebo group, respectively.

***Conclusions:*** The addition of prophylactic oral broad-spectrum antimicrobial therapy to the standard antepartum management of twin gestations had no demonstrable effect on the gestational age at delivery, birth weight, or neonatal complications. There did not appear to be any beneficial effect of the prophylactic use of amoxicillin/clavulanic acid in this clinical setting. © 1995 Wiley-Liss, Inc.

Key words

Multiple gestation, infection, prophylactic antibiotics

Multiple gestation is an important risk factor for preterm labor and delivery with the rate of preterm delivery 2–5 times greater than the rate of singleton pregnancies.[1] The etiology of preterm birth is unknown, although recent reports have implicated infection as a possible cause.[2–5] Randomized clinical trials using antibiotics in hospitalized women with singleton gestations and preterm labor have suggested a significant prolongation in gestation in the treated groups.[3–5] Women randomized to a course of erythromycin or placebo as adjunctive therapy to tocolysis also showed strong statistical trends toward larger birth weights, reduced nursery days, and reduced maternal readmissions for preterm labor in the drug-treated women.[3]

In addition, it has been hypothesized that mechanical forces in twin pregnancy may cause premature cervical dilation which may increase the likelihood of ascending infections. Romero and coworkers[6] found that 11.9% of amniotic-fluid cultures from twin gestations with preterm labor were positive. The purpose of this randomized, controlled trial was to study the effect of prophylactic antibiotic treatment in twin gestations.

Address correspondence/reprint requests to Dr. Mark T. Peters, Department of Obstetrics and Gynecology, 319 West, 1600 South Andrews Avenue, Fort Lauderdale, FL 33316.

Received April 19, 1995
Accepted September 6, 1995

### SUBJECTS AND METHODS

### Study Population

The subjects for this study were selected from the population of women presenting for obstetrical care at Parkland Memorial Hospital, Dallas, TX, between November 1, 1989, and December 1, 1992. These women, all residents of Dallas County, were determined to be economically disadvantaged when they presented for prenatal care at one of the satellite prenatal clinics.

The study was approved by the institutional review board at the University of Texas Southwestern Medical Center. All twin pregnancies diagnosed antenatally in the Parkland Memorial Hospital clinic system were referred to the twin clinic for further evaluation and prenatal care. A patient was considered for enrollment if the estimated gestational age was <33 weeks. The criteria for exclusion were fetal structure abnormalities and monoamnionicity or twin intrapair weight discordancy >25% by ultrasound. In addition, a patient was excluded if she had a medical complication such as hypertension, diabetes, hyperthyroidism, or cardiac disease or if she had an allergy to penicillin. A patient was also excluded if she was unable to give informed consent in either English or Spanish. Women who met the study criteria were asked to participate, and written informed consents in English or Spanish were obtained.

### Experimental Design

The patients were divided into 2 subgroups based upon gestational age at entry in the study. The patients in the first subgroup were enrolled ≤24 weeks gestation. These patients were randomized to receive either amoxicillin, 500 mg/clavulanic acid, 125 mg, or placebo (vitamin C, 500 mg) every 8 h for 7 days in their 24th week of pregnancy and again in their 28th week of pregnancy. Those patients who were enrolled >24 weeks but <33 weeks gestation were randomized to receive a single week-long course of amoxicillin/clavulanic acid or placebo regimen either at the 28th week or at the time of enrollment (up to 32 weeks). This subgrouping was necessary because our obstetrical clinics do not employ "universal sonography," resulting in many cases of twins not being diagnosed until the third trimester. The drug was administered in a double-blinded fashion. A patient's compliance was assessed by counting the number of pills left at the following clinic visit. She was considered compliant if she had taken more than half of her pills.

The standard protocol for following all twin gestations in this clinic was not changed for the management of the study patients. Specifically, the prenatal laboratory work for a patient presenting to a neighborhood obstetrical clinic in Dallas County included a cervical *Neisseria gonorrhoeae* culture, a urine culture, and syphilis serology. After the diagnosis of twins was made, the patient was referred to a centralized clinic at Parkland Memorial Hospital. The prenatal care at the centralized clinic consisted of 1) weekly visits after 22 weeks gestation including digital cervical examinations performed by an attending staff member or resident, 2) ultrasound examinations at 3–4-week intervals, 3) iron and folate supplementation, and 4) hospitalization for medical or obstetrical indications such as preeclampsia, hydramnios, or >25% discordance of estimated fetal weight on ultrasound. Additionally, any woman presenting with preterm labor or a cervical score (cervical dilation in cm subtracted from the cervical length in cm) <0 on her clinic cervical examination was admitted to labor and delivery at Parkland Memorial Hospital.[7] If not in active labor on admission, she was hospitalized at bed rest until her 32nd week of gestation. No tocolytics or steroids for fetal lung development were used. The time of delivery was determined by medical or obsterical indications. In the absence of complications, she was delivered electively between 39 and 40 weeks gestation. The route of delivery was decided by the responsible staff physician when the woman presented to the labor and delivery area. The data regarding pregnancy outcomes were abstracted from the hospital charts of the mothers and neonates.

### Statistical Analysis

A power analysis was performed prospectively. Assuming a preterm delivery rate of 50% (≤36 weeks) and assuming that all preterm deliveries were caused by infection, we determined that 45 patients would be required in each group to demonstrate a halving of the preterm delivery rate to 25% with 80% power and an $\alpha$ error at the 5% significance level. Prospectively assuming a 25–50% noncompliance factor, we planned to enroll 75 patients per group to account for noncompliance. A comparison of the compliance of the groups was made using Fisher's

**TABLE 1.** Maternal demographics

| | Amoxicillin/clavulanic acid | | | Placebo | | |
|---|---|---|---|---|---|---|
| Characteristics | Total | 24-week entry | 28-week entry | Total | 24-week entry | 28-week entry |
| No. of subjects (%) | 76 (100) | 41 (100) | 35 (100) | 73 (100) | 36 (100) | 37 (100) |
| Mean age (years) | 25.4 | 25.5 | 25.3 | 25.5 | 25.4 | 25.5 |
| Gravidity >1 (%) | 57 (75) | 31 (76) | 26 (74) | 58 (80) | 28 (78) | 30 (81) |
| Race | | | | | | |
| Black (%) | 27 (36) | 15 (37) | 12 (34) | 26 (36) | 13 (36) | 13 (35) |
| White (%) | 19 (25) | 13 (32) | 6 (17) | 13 (17) | 6 (17) | 7 (19) |
| Hispanic (%) | 29 (38) | 12 (29) | 17 (49) | 34 (47) | 17 (47) | 17 (46) |
| Oriental (%) | 1 (1) | 1 (2) | 0 (0) | 0 (0) | 0 (0) | 0 (0) |

exact test. The groups were compared for maternal age with the Student's t-test, for gravidity with the exact linear-by-linear trend test, and for race or ethnicity using the (2 × 4) Fisher's exact test. The birth weights were compared using a repeated-measures analysis of variance that included a within-subjects effect for the twins. A nursery stay >6 days was evaluated using a repeated-measures strategy for categorical data. The incidence of respiratory distress syndrome and neonatal sepsis was evaluated using chi-squared analysis.[8]

### RESULTS

During the 37-month study, 149 patients with twin pregnancies were enrolled, with 76 receiving amoxicillin/clavulanic acid and 73 receiving placebo. Seventy-seven were randomized <24 weeks gestation and given 2 courses of drugs. Of these 77 patients, 41 received amoxicillin/clavulanic acid and 36 received placebo. In the 72 patients randomized >24 weeks gestation (28–32 weeks), 35 patients received amoxicillin/clavulanic acid and 37 received placebo. Eight patients were lost to follow-up, 5 in the drug group and 3 in the placebo group. No maternal or neonatal data were available for 6 who delivered elsewhere. The other 2 were delivered at Parkland Memorial Hospital, but their neonatal records could not be located. Both of these sets of twins were admitted to the normal newborn nursery, presumably with no significant problems. The treatment regimens were generally well tolerated. Thirty patients reported adverse effects including nausea and vomiting (9/30) and diarrhea (9/30). There were no serious adverse effects, although 16 subjects stated they discontinued treatment before completing the course due to side effects. Of these 16 subjects, 7 were taking placebo and 9 amoxicillin. The overall compliance was no different in the drug vs. placebo group (56/76 vs. 50/73) ($P = 0.59$).

The demographic data of the study groups are shown in Table 1. There was no difference in mean age or gravidity ($P = 0.33$) in the groups. The racial distribution was similar for the groups ($P = 0.57$).

The pregnancy outcomes are summarized in Tables 2 and 3. One patient in the placebo group had an unexplained antepartum demise of both twins at 35 weeks. There were 2 neonatal deaths in the amoxicillin/clavulanic acid group. The first death occurred in a twin gestation delivered at 30 weeks (1,695-g male). This infant died on day-of-life 12 from sepsis, being diagnosed as having congenital leukemia. The sibling was a 1,535-g female who did well. The other neonatal death was a small-for-gestational-age, 1,960-g male delivered at 36 weeks who died on day-of-life 4. The cause of death was necrotizing enterocolitis due to a midgut volvulus that resulted in a complete bowel obstruction. This infant's cotwin, also small for gestational age, was a 1,920 g-male who was noted to have chordee. Nine neonates with normal cotwins had structural abnormalities. Table 2 illustrates the outcome data including the numbers of patients delivering at various gestational ages. The mean gestational ages at delivery in the drug and placebo groups were similar (35.9 weeks and 35.7 weeks, respectively). No significant difference in the rate of delivery <30 weeks was found in the 2 groups (5/76 vs. 4/73, $P = 0.75$); the 95% confidence intervals for the groups were 2.3–15.7% and 1.6–14% for the drug and placebo groups, respectively. We also found no significant difference in the rate of delivery <34 weeks in the 2 groups (14/76 vs. 19/73, $P = 0.34$); the 95% confidence intervals were 11.2–30.9%

TABLE 2. Maternal outcome

| Outcome | Amoxicillin/clavulanic acid | | | Placebo | | |
|---|---|---|---|---|---|---|
| | Total (N = 71) | 24-week entry (N = 39) | 28-week entry (N = 32) | Total (N = 72) | 24-week entry (N = 36) | 28-week entry (N = 36) |
| Mean gestational age at delivery (weeks) | 35.9 | 35.6 | 36.2 | 35.7 | 35.4 | 35.7 |
| 25–28 weeks (%) | 0 (0) | 0 (0) | 0 (0) | 2 (3) | 2 (6) | 0 (0) |
| 29–30 weeks (%) | 5 (7) | 3 (8) | 2 (6) | 2 (3) | 1 (3) | 1 (3) |
| 31–33 weeks (%) | 7 (10) | 3 (8) | 4 (12) | 8 (11) | 3 (8) | 5 (14) |
| 34–36 weeks (%) | 24 (34) | 16 (41) | 8 (24) | 33 (46) | 17 (47) | 16 (44) |
| 37–39 weeks (%) | 31 (44) | 17 (44) | 14 (44) | 25 (35) | 13 (36) | 12 (33) |
| >40 weeks (%) | 4 (6) | 0 (0) | 4 (12) | 2 (3) | 0 (0) | 2 (6) |
| Prematrue rupture of membranes (%) | 7 (10) | 5 (13) | 2 (6) | 4 (6) | 2 (6) | 2 (6) |
| Chorioamnionitis (%) | 1 (1) | 0 (0) | 1 (3) | 4 (6) | 2 (6) | 2 (6) |
| Cesarean delivery (%) | 46 (65) | 23 (59) | 23 (72) | 43 (60) | 26 (72) | 17 (47) |

TABLE 3. Neonatal outcome

| Outcome | Amoxicillin/clavulanic acid | | | Placebo | | |
|---|---|---|---|---|---|---|
| | Total (N = 142) | 24-week entry (N = 78) | 28-week entry (N = 64) | Total (N = 140) | 24-week entry (N = 70) | 28-week entry (N = 70) |
| Mean birth weight (g) | 2,358 | 2,323 | 2,401 | 2,344 | 2,362 | 2,325 |
| 750–1,000 (%) | 0 (0) | 0 (0) | 0 (0) | 3 (2) | 3 (4) | 0 (0) |
| 1,001–1,500 (%) | 7 (5) | 5 (6) | 2 (3) | 6 (4) | 3 (4) | 3 (4) |
| 1,501–2,000 (%) | 24 (17) | 13 (17) | 11 (17) | 26 (19) | 10 (14) | 16 (23) |
| 2,001–2,500 (%) | 53 (37) | 30 (39) | 23 (36) | 49 (35) | 22 (31) | 27 (39) |
| ≥2,501 (%) | 58 (41) | 30 (39) | 28 (44) | 56 (40) | 32 (46) | 24 (34) |
| Still births[a] (%) | 0 (0) | 0 (0) | 0 (0) | 2 (1) | 0 (0) | 2 (3) |
| Neonatal deaths[a] (%) | 2 (1) | 1 (1) | 1 (2) | 0 (0) | 0 (0) | 0 (0) |
| Mean hospital stay (days) | 9.8 | 10.8 | 8.7 | 11.5 | 12.4 | 10.5 |
| Hospital stay ≥7 days (%) | 54 (38) | 37 (47) | 17 (27) | 56 (40) | 29 (41) | 27 (39) |
| Respiratory distress syndrome[b] (%) | 8 (6) | 3 (4) | 5 (8) | 6 (4) | 2 (3) | 4 (6) |
| Sepsis (%) | 5 (4) | 3 (4) | 2 (3) | 4 (3) | 2 (3) | 2 (3) |

[a]See text for details.
[b]Requiring intubation and diagnosed by neonatologist.

and 17.2–39.1% for the drug and placebo groups, respectively. The rate of premature rupture of the membranes (PROM) before labor and chorioamnionitis was not significantly different in the drug and placebo groups ($P = 0.38$ and 0.37, respectively).

The neonatal outcomes are summarized in Table 3. There was no difference in mean weight in the drug vs. placebo group (2,358 vs. 2,344 g, $P = 0.86$); the 95% confidence intervals were 2,278–2,438 and 2,252–2,435 g, respectively. In addition, there was no difference in length of nursery stay using a cutoff of 6 days ($P = 0.76$). The overall incidence of respiratory distress syndrome was no different (8/142 vs. 5/140, $P = 0.57$). The incidence of neonates with sepsis was also similar in the drug vs. placebo group (5/142 vs. 4/140, $P = 1.07$). The perinatal mortality rate was 14.2 (4/282).

### DISCUSSION

Preterm labor is the leading cause of perinatal morbidity and mortality. In twin gestations, the perinatal mortality is 4-fold that of singleton gestations mainly because of an increased incidence of preterm labor.[1,2] Various attempts have been made to decrease this incidence including prophylactic bed rest, cervical cerclage, various tocolytic agents, and home uterine activity monitoring.[9–16] None has shown any clear beneficial effects although the rate of preterm delivery has been found to be decreased with home uterine activity monitoring.[9,10] Some investigators have suggested that this effect may be due to frequent contact with health-care providers, not to the monitoring device.[17] Recently, a reduction in very low birth weight deliveries and perinatal mortality in a specialized, multidisciplinary twin clinic was reported.[18]

The cause of preterm labor is unknown. Recent studies suggest a link between subclinical infection and prematurity.[2] There have been a variety of cervicovaginal, upper-genital-tract, and amniotic-fluid microorganisms associated with preterm delivery.[3]

Antibiotic intervention trials have been conducted in antepartum singleton pregnancies with high-risk factors for preterm delivery.[19–23] A study of patients with positive cervical cultures for *Mycoplasma* treated with erythromycin revealed a significantly lower rate of PROM.[19] In 2 studies of patients with positive chlamydial cultures who were treated, the rates of preterm labor, small-for-gestional-age infants, and PROM were lower.[21,22] Other studies using antibiotics as an adjunct to tocolysis in patients in preterm labor have shown a delay in delivery, but no statistically significant decrease in perinatal mortality or increase in birth weight.[4,23–25] Whether or not an increase in the length of gestation has any long-term benefit for the infant is unknown. In patients with positive vaginal-swab cultures for *M. hominis* or *Ureaplasma urealyticum,* treatment with erythromycin resulted in a statistically significant greater birth weight.[20]

It has been hypothesized that mechanical forces in twin pregnancy may cause premature cervical dilation leading to an increased likelihood of ascending infections. Romero and coworkers[6] found that 11.9% of amniotic-fluid cultures in twin gestations with preterm labor were positive. These patients had a high incidence of respiratory distress syndrome and lower mean birth weight.

The pregnancy outcomes of the present study compare favorably with those of recently published studies of twin pregnancies.[10,18] Since neither tocolysis nor home uterine monitoring was employed in our study, the frequent patient contact by health-care providers, as suggested by others, may indeed have had a beneficial effect.[9,10,18]

The present study demonstrated no effect of the antibiotic regimen on prolongation of pregnancy or neonatal outcome. It is possible that a different or longer antibiotic regimen may have shown a difference. In addition, our observations in an economically disadvantaged population might differ from those in a more stable, middle-class population.

Since the assumption that all preterm labor is caused by infection is probably not true in all cases, as demonstrated by Romera et al.,[6] using this assumption in the power calculation may not rule out a $\beta$ error. Since no microorganism tests were obtained for this study, more specific tests for bacterial infections might result in identifying patients at higher risk for preterm labor caused by infection.

The potential problems with compliance were considered in determining the treatment regimen. In the 6-week erythromycin protocol from Brigham and Women's Hospital, fewer than half of their patients were compliant.[17] The changes in normal bacterial flora resulting in fungal overgrowth and bacterial resistance make long antibiotic therapy impractical and potentially harmful. Amoxicillin/clavulanic acid was chosen for its broad-spectrum coverage, its safety in pregnancy, and its better toleration compared with erythromycin.

Based on the results of this study, the routine prophylactic use of amoxicillin/clavulanic acid to prevent preterm delivery in twins cannot be recommended.

### ACKNOWLEDGMENTS

We gratefully acknowledge the invaluable assistance of Carolyn Quan, M.D., Debra Eckerly, M.D., and Michelle Clement, M.D. We express deep appreciation to the housestaff at Parkland Memorial Hospital for their tireless care of patients and to Linda K. Jordan for her expert editorial assistance.

### REFERENCES

1. Kleinman JC, Fowler MG, Kessel SS: Comparison of infant mortality among twins and singletons: United States, 1960 and 1983. Am J Epidemiol 133:133–143, 1991.
2. Romero R, Mazor M, Wu YK, et al.: Infection in the pathogenesis of preterm labor. Semin Perinatol 262–279, 1988.
3. Gibbs RS, Romero R, Hillier SL, et al.: A review of premature birth and subclinical infection. Am J Obstet Gynecol 166:1515–1528, 1992.
4. McGregor JA, French JI, Reller LB, et al.: Adjunctive erythromycin treatment for idiopathic preterm labor: Results of a randomized, double-blind, placebo-controlled trial. Am J Obstet Gynecol 154:98–103, 1986.

5. McGregor JA, French JI, Richter R, et al.: Cervicovaginal microflora and pregnancy outcomes: Results of a double blind, placebo-controlled trial of erythromycin treatment. Am J Obstet Gynecol 163:1580–1591, 1990.
6. Romero R, Shamma F, Avila C, et al.: Prevalence, microbiology, and clinical significance of intraamniotic infection in twin gestations with preterm labor. Am J Obstet Gynecol 163:757–761, 1990.
7. Neilson JP, Verkuyl DAA, Crowther CA, et al.: Preterm labor in twin pregnancies: Prediction by cervical assessment. Obstet Gynecol 72:719–723, 1988.
8. Zar JH: Biostatistical Analysis, 2nd ed. Englewood Cliffs, NJ: Prentrice-Hall, 1984.
9. Knuppel RA, Lake MF, Watson DL, et al.: Preventing preterm birth in twin gestation: Home uterine activity monitoring and perinatal nursing support. Obstet Gynecol Suppl:24–27, 1990.
10. Dyson DC, Crites YN, Ray DA, Armstrong MA: Prevention of preterm birth in high risk patients: The role of education and provider contact versus home uterine monitoring. Am J Obstet Gynecol 164:756–762, 1991.
11. Gilstrap LC III, Hauth JC, Hankins GDV, et al.: Twins: Prophylactic hospitalization and ward rest at early gestational age. Obstet Gynecol 69:578–581, 1987.
12. Ashworth MF, Spooner SF, Verkuyl DAA, et al.: Failure to prevent preterm labor and delivery in twin pregnancy using prophylactic oral salbutamol. Br J Obstet Gynaecol 97:878–882, 1990.
13. Skjaerris J, Aberg A: Prevention of prematurity in twin pregnancy by orally administered terbutaline. Acta Obstet Gynaecol Scand 108 (Suppl):39, 1982.
14. O'Conner MC, Murphy H, Dalymple IJ: Double blind trial of ritodrine and placebo in twin pregnancy. Br J Obstet Gynaecol 86:706, 1979.
15. Dor J, Shalev J, Mashiach S, et al.: Effective cervical suture of twin pregnancies diagnosed ultrasonically in the first trimester following induced ovulation. Gynecol Obstet Invest 13:55, 1982.
16. Sinha DP, Nandalsumar VC, Brough Ak, et al.: Relative cervical incompetence in twin pregnancy: Assessment and efficacy of cervical suture. Acta Genet Med Gemellol (Roma) 28:327, 1979.
17. Merkatz RB, Merkatz IR: The contributions of the nurse and the machine in home uterine activity monitoring system. Am J Obstet Gynecol 164:1159–1162, 1991.
18. Ellings JM, Newman RB, Hulsey TC, et al.: Reduction in very low birth-weight deliveries and perinatal mortality in a specialized, multidisciplinary twin clinic. Obstet Gynecol 81:387–391, 1993.
19. Eschenbach DA, Nugent RP, Rao AV, et al.: A randomized placebo-controlled trial of erythromycin for the treatment of *Ureaplasma urealyticium* to prevent premature delivery. Am J Obstet Gynecol 164:733–742, 1991.
20. McCormack WM, Rosner B, Lee Y, et al.: Effect on birth weight of erythromycin treatment of pregnant women. Obstet Gynecol 69:202, 1987.
21. Cohen J, Valle JC, Calkins BM: Improved pregnancy outcome following successful treatment of chlamydial infection. JAMA 263:3160–3163, 1990.
22. Ryan GM Jr, Abdella TN, McNeely SG, et al.: *Chlamydia trachomatis* infection in pregnancy and effect of treatment on outcome. Am J Obstet Gynecol 162:34–39, 1990.
23. Newton ER, Dinsmor MJ, Gibbs RS: A randomized blinded, placebo-controlled trial of antibiotics in idiopathic preterm labor. Obstet Gynecol 74:562, 1989.
24. Morales WJ, Angel JL, O'Brien WF, et al.: A randomized study of antibiotic therapy in idiopathic preterm labor. Obstet Gynecol 72:829, 1988.
25. Christmas JT, Cox SM, Gilstrap LC, et al.: Expectant management of preterm rupture to delivery. Obstet Gynecol 80:759–762, 1992.
